# Protein and Peptide Substances in the Treatment of Respiratory Syncytial Infection: Current State

**DOI:** 10.3390/molecules27072263

**Published:** 2022-03-31

**Authors:** Anna A. Shtro, Galina D. Petukhova, Aleksandra S. Romanova

**Affiliations:** Smorodintsev Research Institute of Influenza, Professora Popova Str. 15/17, 197376 Saint Peterburg, Russia; gala.iem@gmail.com (G.D.P.); hjvfyjdf2010@yandex.ru (A.S.R.)

**Keywords:** respiratory syncytial virus, antivirals, viral diseases, respiratory diseases, childhood infections

## Abstract

Respiratory syncytial virus infection (RSVI) is an acute medical and social problem in many countries globally. Infection is most dangerous for infants under one year old and the elderly. Despite its epidemiological relevance, only two drugs are registered for clinical use against RSVI: ribavirin (approved in a limited number of countries due to side effects) and palivizumab (Synagis), which is intended only for the prevention, but not the treatment, of infection. Currently, various research groups are searching for new drugs against RSV, with three main areas of research: small molecules, polymeric drugs (proteins and peptides), and plant extracts. This review is devoted to currently developed protein and peptide anti-RSV drugs.

## 1. Introduction

Human respiratory syncytial virus (RSV, hRSV) is one of the most common viral pathogens affecting the respiratory system. This virus is a member of the *Orthopneumovirus* genus (family *Pneumoviridae*) and contains a single-stranded nonsegmented negative-sense RNA genome. There are 10 open reading frames (ORFs) in its genome, which encode 11 proteins. They are: one membrane protein (M1), two non-structural proteins (NS1 and NS2), two nucleocapsid proteins (N and P), three surface proteins coating the virion—fusion (F), small hydrophobic (SH) and attachment (G), and an M2 gene which contains two ORFs, resulting in the production of M2.1 and M2.2, and large (L) protein [1].

F and G proteins are two major immunogenic proteins. The fusion protein, F, can exist in a prefusion form (pre-F) that undergoes a conformational change to post-F after the membrane fusion. This protein is the target of most neutralizing antibodies and, thus, is used for the most monoclonal antibody preparations and vaccine development [2].

The attachment glycoprotein, G, also plays important roles in RSV infection and in the damage of host immunity. It is less immunogenic than the F protein, eliciting approximately 2 to 10% of human serum neutralizing antibodies [3]. However, the G protein contains a central conserved domain, or nonglycosylated region, that is nearly invariant across different strains of RSV, which is the target of broadly neutralizing antibodies [4].

Due to the variety and severity of clinical manifestations in young children, RSV infection remains a significant medical and social problem today [5,6,7]. In infants and children in the first years of life, RSV infection can cause severe bronchiolitis or even death. Vulnerable groups in relation to RSV are, also, people over 65 years of age, in whom the infection leads to increased hospitalization and mortality, and patients with weakened immunity. The risk of serious illness in adults is increased by the presence of chronic respiratory diseases or circulatory disorders and is associated with a higher viral load.

In children under five years of age, RSV causes more than 30 million cases of acute lower respiratory tract infection per year; 3.2 million of them are severe and require hospitalization [8]. Among children hospitalized with RSV infection, mortality averages 1%, but reaches 37% with a burdened history [9]. In children under three years of age, RSV is the cause of 50–90% of bronchiolitis, 5–40% of pneumonia, and 10–30% of tracheobronchitis. A complicated clinical course is most often recorded in children aged 6 weeks to 9 months [5,10,11]. In 2015, there were about 1.5 million episodes of RSV in older people in industrialized countries (data for developing countries were not available). Of these, approximately 14.5% (214,000) were hospitalized [12].

In 2020, in the context of quarantine and preventive measures aimed at preventing the spread of COVID-19, there were changes in RSV outbreak seasonality. In particular, there was a decrease in the incidence of RSV, as well as other seasonal respiratory viruses in China [13] and Australia [14]. Nevertheless, a 2020 meta-analysis of thirty studies, including 3834 patients, showed that RSV continued to circulate in parallel with SARS-CoV-2 and could join as a concomitant infection in some hospitalized patients. Its authors noted the presence of RSV/bacterial coinfections (7%) and RSV/viral coinfections (3%), with RSV/influenza A representing the most common viral coinfection [15].

Quarantine measures have led to a lack of RSV immunity in children born in 2020–2021. This is associated with a surge in RSV infection in the United States [16] and Japan [17] in July 2021. Data for other countries is not yet widely available, but a survey carried out by our institute (data not yet published) shows the same trend. Thus, due to the widespread prevalence of infection and the possibility of severe consequences, the question of finding a means of prevention and antiviral therapy for RSV infection remains relevant and is currently under active study.

Many attempts to obtain an anti-RSV vaccine have been made, but they have not been successful. Research work continues and four new vaccines are in phase III trials. Of the four RSV vaccines, two, from GSK and Pfizer, contain the stabilized pre-F protein itself. Another, from Janssen, uses a modified adenovirus that produces pre-F after delivery into the body, plus a dose of the pure protein, in the same shot. The fourth, from Moderna, delivers modified mRNA that produces pre-F once the RNA is inside cells. Preliminary results are encouraging, but the study is not yet complete [18].

Thus, despite decades of research into RSV biology, safe and effective vaccines against this infection have not yet been registered.

The broad-spectrum antiviral compound ribavirin and the passive immunoprophylaxis agent palivizumab (Synagis^®^) remain approved for use [19].

Ribavirin, a broad-spectrum antiviral compound, is a synthetic analogue of guanosine which was FDA approved for the treatment of RSV infection in 1986. At the molecular level, it stops the synthesis of viral RNA. Due to its significant toxicity, its use for RSV infection is recommended mainly in seriously ill infants from high-risk groups [17,20]. To date, ribavirin remains the only pathogen targeting therapy [20,21].

Palivizumab is used in passive immunoprophylaxis against RSV infection and is a humanized monoclonal antibody (mAb). Palivizumab has been shown to significantly reduce the severity of illness in high-risk children [22]; it also reduces RSV hospitalization in infants [23]. However, prophylaxis with palivizumab is inaccessible to many patients due to its high cost and complex administration schedule, especially in developing countries. In addition, palivizumab is usually prescribed for children with predetermined risk factors [24]; some of these go undetected in susceptible children [25].

In this context, there is an urgent need for an active search for preventative and therapeutic means of therapy related to RSV infection. As such, many laboratories around the world are engaged in the development of compounds for this illness. Conventionally, all developed compounds can be divided into three large groups: (1) low molecular weight compounds of various origins, (2) high molecular weight compounds (primarily protein), and (3) various plant extracts and mixtures. This review focuses on the second group: high molecular weight compounds currently being developed, primarily of a protein or peptide nature. These substances can be conditionally subdivided into the following categories: monoclonal antibodies, nanoantibodies, and oligopeptide compounds of natural or synthetic origin.

## 2. Monoclonal Antibodies

Apart from vaccines, the most promising group of compounds for the prevention of respiratory syncytial infection are monoclonal antibodies. When administered to a patient according to a specific regime, they remain in circulation.

Currently, the clinicaltrials.gov website has registered a total of more than 300 studies on the subject of RSV infection [26]. According to PATH (RSV Clinical Trial Tracker) [27], 119 studies of protein compounds have currently been registered, the overwhelming majority of which relate to anti-RSV vaccines. Monoclonal antibodies account for 11 studies (2014–2020) devoted to anti-F mAbs: MK-1654 (Merck, Darmstadt, Germany), REGN2222 (Regeneron Pharmaceuticals, Tarrytown, NY, USA), and MEDI8897 (AstraZeneca, Cambridge, UK/Sanofi Pasteur Paris, France collaboration). Presently, another four mAb preparations are in the preclinical study stage [28]: three anti-F mAbs (Aridis Pharmaceuticals, Los Gatos, USA, Gates MRI, USA, and mAbxience/UCAB, Madrid, Spane collaboration) and one anti-N mAb (Pontificia Universidad Católica de Chile).

The only licensed preparation for this purpose to date is palivizumab (Sinagis), a humanized IgG_1k_ monoclonal antibody that interacts with the A epitope of the fusion protein (F) antigen. The palivizumab molecule consists of human (95%) and mouse (5%) sequences. The preparation was first approved for use in the United States in 1998 [28].

The administration regime consists of five intramuscular injections carried out at one-month intervals during the seasonal rise in RSV incidence (October/November to March). Use of the preparation is very expensive, and its effectiveness has been proven only in a preventive context. Palivizumab exhibits a pronounced neutralizing and inhibitory effect on fusion proteins of subtype A and B RSV strains. Prophylactic administration of palivizumab to newborns during the epidemic season led to a significant decrease in the frequency of hospitalization of patients with RSV infection [23]. However, due to the very high cost [19] of treatment ($20,000 per child over 4–5 doses during the epidemic season) and limited efficacy in children born after 29 weeks of gestation, palivizumab was discontinued in children in this group [29,30,31].

Evidence suggests that palivizumab has some therapeutic effects. Safety and efficacy results for palivizumab, summarized in a systematic review by Canadian researchers, [32] suggest that it is promising for further clinical trials as a therapy. The data combined by the authors indicate that 3 out of 25 patients with upper respiratory tract infections, and 5 out of 88 patients with lower respiratory tract infections, who received palivizumab died from RSV. The required palivizumab concentrations were maintained for at least three weeks after intravenous injection at a dose of 15 mg/kg. The use of palivizumab led to a significant decrease in the RSV content in tracheal secretions (but not in nasal lavage) on days one and two of therapy [33].

There have been many attempts to create a more effective, longer acting, and less expensive analogue of palivizumab. A number of candidate mAb studies, which have not yet reached the stage of clinical trials, have been published. Binding to different F protein epitopes has been shown to be effective against RSV in vitro and/or in vivo. Palivizumab (MEDI-493) appeared in the process of searching for solutions to increase the effectiveness of the RSV-IGIV, a polyclonal human Ab preparation for intravenous infusion. Felvizumab (RSHZ19), also a humanized mAb, was developed for the same purpose [34].

A comparative study showed that palivizumab significantly reduced the number of hospitalizations with a diagnosis of RSV, while felvizumab did not show significant efficacy [31]. To determine if different clinical results were associated with differences in biological activity, additional comparative studies in vitro and in vivo were carried out. Palivizumab was four- to five-fold more effective than felvizumab in binding antigens, neutralizing RSV, and inhibiting fusion. Although both preparations were effective against RSV subtypes A and B in the cotton rat model, two- to four-fold higher doses of felvizumab were required for similar protection. Felvizumab (RSHZ19) studies appear to have been discontinued at this time [34].

Motavizumab (Numax, MEDI-524), a humanized second-generation mAb from AstraZeneca, has become one of the most promising developments in the F protein binding mAb group [35]. Motavizumab differs from palivizumab by 13 amino acid residues, and it also targets the viral F protein. The preparation showed good results in an experiment with BALB/c mice intranasally infected with RSV A2. Palivizumab or motavizumab was administered once: 24 h before or 48 h after RSV inoculation. Regardless of the time of administration, viral loads in bronchoalveolar lavage samples were significantly reduced. The amount of virus in the lungs of mice on days 5 and 28 after infection was significantly reduced only when motavizumab was used one day before the introduction of the virus. The same scheme showed the best results when analyzing the histopathological portrait in the lungs; airway obstruction and post-methacholine airway hyperresponsiveness were significantly reduced in mice in this group compared with the other groups and controls. Motavizumab was superior to palivizumab in reducing viral replication as measured by inflammatory and clinical markers of illness severity and its effect on long-term pulmonary abnormalities [36].

More than ten motavizumab clinical trials have been conducted, including phases I to III [37], with quite promising results. It was well tolerated, did not cause significant side effects, and was similar in pharmacokinetics to palivizumab (five doses). It reduced the number of RSV hospitalizations by 83% compared with the placebo and 26% compared with palivizumab [38]. It also reduced the incidence of lower respiratory tract secondary infections in outpatients. However, the FDA finally refused to register motavizumab due to a lack of advantages over palivizumab [39], as well as due to the discovered side effects in the form of allergic reactions [40,41].

Palivizumab and motavizumab recognize an F protein epitope that is unchanged during the fusion process; they do not prevent its initial conformational changes. Further searching for effective mAbs was carried out towards the development of those capable of blocking the F protein before the fusion stage. Antibodies that can lock F protein conformation into its prefusion state have increased efficiency and longer serum half-lives [19].

These compounds include nirsevimab (MEDI-8897, AstraZeneca, Cambridge, UK), which recognizes the antigenic site Ø [42]. Due to the described advantages, a single MEDI8897 injection is sufficient for protection during the epidemic season, as opposed to four or five needed with previous generation preparations. In healthy, premature infants, a single nirsevimab injection contributed to a decrease in the number of hospitalizations with RSV, compared with the placebo, during the entire epidemic season [43]. Specifically, 70.1% fewer cases of RSV-associated lower respiratory tract infection were detected with nirsevimab prophylaxis than with the placebo: 2.6% (25 infants) versus 9.5% (46 infants) (*p* < 0.001). The hospitalization rate was 78.4% lower: 0.8% (8 infants) versus 4.1% (20 infants) (*p* < 0.001). These differences were consistent over the 150-day post-dose period across geographic regions and across RSV subtypes. RSV-neutralizing activity and the ability to maintain protection against RSV during the 5-month epidemic season, after a single intramuscular injection, were shown [44]. Thus, the preparation was safe and could protect healthy, premature babies from RSV. AstraZeneca is currently in phase III clinical trials with nirsevimab [45].

MK-1654 (Merck, Darmstadt, Germany), another mAb targeting F protein, has shown pronounced efficacy in the cotton rat RSV model. Prophylactic administration showed strong, dose-dependent antiviral activity (RSV-A, RSV-B) in the lungs and nasal passages (material taken 4 days after infection). Lung EC_50_ values were 1.1 μg/mL and 1.9 μg/mL for RSV-A and RSV-B. In the upper respiratory tract, these values were 9.9 μg/mL and 8.5 μg/mL, respectively. The first phase of clinical trials showed that the overall safety profile of MK-1654 was similar to that of the placebo, and there were no side effects [46]. In 2019, it was shown to be ineffective in a clinical trial [47].

Another preparation, suptavumab (REGN-2222, Regeneron Pharmaceuticals NY, USA), specific for antigenic site V, successfully reached phase III clinical trials, but did not pass due to insufficient efficacy in healthy, premature infants [48]. In the last decade, researchers’ attention has switched to mAbs directed at the G protein. Several experimental studies have been carried out on the G protein’s roles in viral penetration, virus neutralization, and RSV-mediated pathology. In an experiment in mice, data were obtained using mouse mAb 131-2G (a reagent for enzyme immunoassays sold by such manufacturers as Merck) against the G protein. In RSV-infected mice, it caused reductions in weight loss, the number of cells in bronchoalveolar lavage, reactivity of respiratory pathways, and Th2 cytokine production. These effects were faster than palivizumab therapy [49].

The prophylactic and therapeutic administration of two mAbs specific to the G protein, 2B11 and 3D3, have also been studied in a model of RSV infection in BALB/c mice. Both anti-G mAbs reduced viral load, leukocyte infiltration, IFN-γ expression, and IL-4 expression in cell-free supernatants in bronchoalveolar lavage. This makes such compounds promising candidates for the prevention and treatment of RSV [50].

## 3. Nanoantibodies

Preparations developed based on nanoantibodies can be distinguished into a separate subgroup. Nanoantibodies are isolated variable domains of heavy-chain antibodies (aka VHH “single-domain antibodies”) that can function in the absence of other domains [51]. Such antibodies have a number of advantages over classical mAbs, including: their small size (15 kDa), which facilitates penetration into cells and tissues; flexibility of hypervariable regions, which facilitates access to previously inaccessible epitopes; high solubility; thermal stability; resistance to extreme pH; easy additional modification to enhance therapeutic and diagnostic potential; and cost-effective production in bacterial systems. Thanks to these features, nanoantibodies have found application in the development of anti-RSV preparations. Currently, all these studies are still at the stage of preclinical trials.

Thus, ALX-0171, a trimeric nanoantibody that binds the F protein antigenic site II, was more active in neutralizing RSV in vitro than palivizumab. It was shown to completely block replication below the detection limit for 87% of the viruses tested, while palivizumab blocked replication for 18% of the viruses at a fixed concentration. ALX-0171 also caused a significant decrease in RSV titers, in both the nose and lungs, when administered prophylactically or therapeutically directly into the lungs of cotton rats. The authors of this study believe that ALX-0171 has significant potential for the treatment of RSV-mediated illnesses [52].

However, a 2018 phase IIb, double-blind, randomized, placebo-controlled study of inhaled ALX-0171 showed different results [53]. The use of ALX-0171 did not affect infection outcome, compared with the placebo, in children hospitalized with RSV lower respiratory tract infection. Picomolar concentrations of another nanobody-based preparation, F-VHHb, protected BALB/c mice from RSV infection and associated pneumonia. Therapeutic administration of these nanobodies after RSV infection reduced viral replication and decreased viral pneumonia [54].

Thus, to date, the search for passive immunoprophylaxis continues with a focus on the synthesis of various types of mAbs or nanoantibodies specific to various viral proteins (mainly F and G). However, none of the candidates have yet been licensed. The closest to this stage is nirsevimab. However, evidence of the presence of escape mutations that allow the virus to escape from therapeutic agents targeting the F or G proteins [55,56,57,58], and the lack of effective yet inexpensive preparations, dictate the need to search for fundamentally new candidate agents for the therapy and prevention of RSV infection. We next consider peptide agents, which can be considered potentially promising in this respect.

## 4. Preparations of Natural Origin

In a separate search direction, it is possible to single out substances of a peptide nature that are not mAbs, with which an antiviral effect against RSV, or a wide range of other viral agents, has been identified. In most cases, these are molecules, or their modified analogs, that somehow participate in the host organism’s defense against viral infection through innate immunity. Despite proven antiviral activity in vitro, the use of such molecules is often limited by their low stability in biological media.

These substances include, for example, viperin, an interferon-induced cellular protein that does not differ much in various groups of animals [59]. It has been shown to inhibit the replication of a wide range of viruses by producing the ribonucleotide 3′-deoxy-3′4′-didehydro-CTP (ddhCTP), which acts as a terminator for viral RNA polymerase. The effect of RSV infection on viperin protein expression in the sensitive HEp2 cell line and the non-permissive RAW 264.7 line has been studied [60]. In HEp2 cells, low levels of this protein were localized in virus-induced inclusion bodies and did not reduce viral spread in the cells. In RAW 264.7 cells, a relatively high level of viperin expression was detected, and it was localized in the cytoplasm of infected cells. In an experiment with transfected HeLa cells expressing viperin, there was no significant inhibitory effect on viral protein expression, and there was no prevention of viral infection. However, although inclusion body formation was not inhibited, early expression of the viperin protein was associated with the inhibition of viral filament formation and reduced cell-to-cell transmission of the virus. Inhibition of viral filament formation has also been observed in HEp2 cells expressing viperin. The authors of this study suggest that the ability of viperin to disrupt RSV transmission by inhibiting viral filament formation may serve as a basis for further study of its activity against this virus.

Another group of authors found anti-RSV activity with cathelicidins, which are cationic peptides expressed in inflamed lungs and which are involved in the host’s innate defense against infection [61]. Human cathelicidin LL-37 has been shown to have anti-RSV activity in vitro. LL-37 prevented virus-induced cell death in epithelial cultures, significantly reduced the formation of new infectious particles, and reduced the spread of infection. The authors of this study conclude that LL-37 may represent an important component of the human body’s innate defense against RSV infection, and prophylactic modulation of LL-37 expression, and/or the use of synthetic analogs after infection, may be a promising strategy for the development of compounds against RSV.

The following potential therapeutic agents against RSV infection are not of a peptide structure, but are inhibitors of protein expression, so we found it necessary to mention them here. Deoxyribozymes’ activity is based on the ability to bind and cleave complementary RNA sequences. It has been shown that deoxyribozyme DZ1133, targeting the conserved genomic RNA sequence of the RSV nucleocapsid protein, is capable of inhibiting the replication of various RSV strains, including those in subgroups A and B [62]. Administration of DZ1133 caused reductions in the viral content in the lungs of BALB/c mice infected with RSV, expression of viral mRNA, airway inflammation, and the number of leukocytes in the bronchoalveolar lavage fluid of animals. The antiviral effect of DZ1133 was dose dependent (0.2–0.8 mg) and more effective than the inhibition of gene expression by antisense oligonucleotides. However, the levels of cytokines induced by RSV infection (TNF-α, IFNγ, IL-12, IL-10) were not affected by DZ1133 treatment. The data obtained make it possible to consider DZ1133 a potential therapeutic agent against RSV infection caused by strains A and B.

In a study of the role of surfactant protein A (SP-A) in antiviral protection in SP-A^−^ mice, it was found that during intratracheal RSV infection, absence of SP-A leads to more severe pulmonary infiltration with polymorphonuclear leukocytes and higher RSV titers (plaque-forming units) in lung homogenates compared with control animals (SP-A^+/+^) [63]. Proinflammatory cytokine levels (TNF-α, IL-6) were also increased in the lungs of SP-A^−^ mice. The co-administration of RSV with exogenous SP-A led to a decrease in viral titers and inflammatory markers in the lungs of SP-A^−^ mice. The authors of this study came to the conclusion that SP-A plays a significant role in protecting the host from RSV infection and that it is promising for further research in the context of therapy for RSV infection. Thus, artificial SP-A may be a good strategy to prevent or treat RSV pulmonary infections.

The cytotoxicity and anti-RSV activity of two recombinant human superoxide dismutase (SOD) variants in tissue culture and in cotton rats have also been studied [64]. These featured manganese (rhuMnSOD) or copper/zinc (rhuCuZnSOD) in the active center. In tissue culture studies, there was no obvious cytotoxicity with either preparation (CC_50_ > 1000 μg/mL), but also no inhibition of the virus. However, in vivo experiments with aerosol administration of either rhuMnSOD or rhuCuZnSOD showed a significant decrease in viral replication in the lungs. The protective effect was dose dependent, and it was not observed with the parenteral (intraperitoneal) or intranasal administration of the SOD compound.

Chemokines, proteins that attract leukocytes to foci of infection, have also been considered as targets in anti-RSV therapy. Some enveloped viruses are known to bind to glycosaminoglycans (GAGs), such as negatively charged heparan sulfate. As such, GAGs are important for initial contact (between virus and host cell) and for cell infection. Research has been carried out [65] showing that the C-terminal peptides CXCL9 and CXCL12γ (chemokine derivatives) have antiviral activity against a number of viruses, including RSV. The authors of this study consider such peptides to be promising molecules for the development of new antiviral agents. Suppression of virus-cell interactions, by blocking GAG binding sites on the host cell, is a possible mechanism of antiviral activity against a wide range of infectious agents, including resistant strains.

## 5. Synthetic Peptides

In a separate direction, one can single out the actively developing in silico search in recent decades and the subsequent synthesis of peptides with the assumed necessary properties. This approach has played a significant role in the search for new synthetic peptides. Screening in silico allows one to study the activity of several thousand preparations without resorting to costly experimental work.

Thus, in the framework of a comprehensive study of synthetic peptides from conserved viral regions (three paramyxoviruses, RSV, human parainfluenza virus type 3, measles virus [66]), a number of such peptides were tested for anti-RSV activity. Peptide preparations showing antiviral activity were purified and tested for their ability to block syncytium formation. One of the peptides, T-118, was most active against RSV; several other peptides from the same group showed moderate activity. The authors of this study believe that the synthesis of such peptides may become a new approach to the development of targeted treatment methods.

A study was also carried out on a synthetic peptide derived from 13 amino acid residues of the F protein, fixed in the alpha-helical conformation [67]. The resulting peptide showed strong anti-RSV activity in vitro in the COS-1 cell line. The authors of this study were the first to show that such peptides can be functional antagonists of important protein–protein interactions.

Another group of authors investigated peptides derived from the F-binding region of the small GTPase RhoA, which can interact with RSV F protein [68]. The RhoA fragment has been shown to be able to block RSV entry into susceptible host cells [69]. It contains a linear peptide sequence corresponding to RhoA amino acids 77–95. Based on the observation that peptide 77–95 can interfere with the binding of F to RhoA in an in vitro enzyme-linked immunosorbent assay, it was initially hypothesized that it inhibits the interaction between the F protein and RhoA, which is required for F-mediated membrane fusion. However, there was no evidence of an interaction between F and RhoA during fusion in vivo. In addition, other agents that should be able to inhibit RhoA–F interaction did not inhibit RSV penetration.

However, based on studies where it was found that synthetic peptide 77–95 inhibits RSV replication in sensitive cells, the authors of this study further showed that RhoA residues 80–90 are sufficient for this activity and a cysteine residue at position 83 plays a crucial role [70]. Peptide 80–94 can form aggregates in, either, reduced or oxidized states. A peptide (83 A), in which the cysteine residue is replaced by alanine, did not form dimers or higher-order aggregates; it did not inhibit RSV replication at any concentration tested. The authors of this study conclude that the formation of peptide multimers derived from RhoA is necessary for the antiviral activity of peptides. They suggest that the observed antiviral activities of these peptides may not be related to the biological functions of their parent molecule.

One of the modern approaches to expanding the possibilities of peptide therapy is the chemical synthesis of peptide dendrimers with a hyperbranched structure. Several groups, including Russian and Italian investigators, have pursued this approach [70,71]. The work of the Italian authors involved the screening of linear, dimeric, and dendrimeric peptides containing clusters of basic amino acids in order to identify peptides capable of binding heparan sulfate (HSPG), the target of HCD on the cell surface, by proteoglycans. The most potent inhibitor of RSV infectivity in this study was the dendrimer SB105-A10 (IC_50_ 0.35 μM and 0.25 μM in Hep-2 and A549 cells, respectively). SB105-A10 was found to bind to both types of cells via HSPG, suggesting that its antiviral activity is, indeed, due to competition with RSV for binding to HSPG on the cell surface. SB105-A10 prevented infection when added, either, before or after virus introduction into cell culture.

The antiviral potential of SB105-A10 was further tested in a pseudo-stratified, highly differentiated model of human airway epithelial tissue obtained by the appropriate culture of human tracheal/bronchial epithelial cells. SB105-A10 strongly reduced the infectivity of RSV in this model and showed no signs of cytotoxicity or proinflammatory effects. The authors of this study consider SB105-A10 to be an attractive candidate for further development as an aerosol agent against RSV infection.

The aim of the study by Russian scientists was to create relatively short linear and dendrimeric cationic peptides and to test their antiviral activity against RSV. Three linear cationic peptides and four peptide dendrimers were synthesized and compared with the known mAbs LL-37 (cathelicidin family) and anti-F0 in terms of cytotoxicity and antiviral activity. Four synthesized peptides showed a cytotoxic effect; two of them were even more cytotoxic than LL-37. The study’s authors attribute this to a combination of a large amount of positive charge and amphipathicity. Non-hydrophobic, dendrimeric peptides did not show cytotoxicity in mammalian cells in the studied concentration range. Two of the seven synthesized peptides, LTP (dendrimer) and SA-35 (linear), had a stronger antiviral effect than the natural LL-37 peptide. Three others showed slightly lower activity than mAbs against F0. The data obtained in that study led its authors to the conclusion that a uniformly-distributed positive charge, with low or moderate amphipathicity, plays a key role in the antiviral activity of the studied peptides.

Recently, the activity of peptides from the N-terminus of phosphoprotein (P) in inhibiting RSV replication by preventing the oligomerization of protein N has been shown [72]. Similar strategies have already been proposed for RSV, rabies virus, and Nipah virus [73,74,75]. However, the authors of previous studies were faced with the problem of the low bioavailability of the obtained peptides. In those studies, the authors used peptide crosslinking technology, which is considered a promising tool for solving this problem. The resulting peptides contain unnatural, olefinic amino acids, which increase the proteolytic stability and cellular permeability of the peptide due to a large, hydrophobic, fully-hydrocarbon macrocycle [76]. The study’s authors screened their RSV protein N-terminal peptides and identified a peptide capable of inhibiting RSV infection in vivo (BALB/c mice), preventing the formation of the NO-P complex (compound **5a**). Those authors conclude that such a strategy for obtaining antiviral peptide compounds is promising [72].

## 6. Conclusions

Decades of RSV research and attempts to create effective vaccines and preparations against this infection have not yet led to the development of optimal antiviral agents. However, they have accelerated progress in studying the pathogenesis of infection and the mechanisms of interaction between the virus and the host cell. The ability of the virus to not only modulate or bypass the host’s immune response, but also form resistance to existing compounds, greatly complicates the task of creating effective preparations against RSV infection. It necessitates new vaccination and therapeutic strategies that are different from our usual approaches.

Apparently, the most promising strategies include in silico search approaches, with the subsequent synthesis and screening of peptide compounds. Their mechanisms of action will in some way be associated with various stages of the virus/cell molecular interaction. Chemical modifications to improve their stability and bioavailability can further improve their potential.

## Data Availability

All data were taken from open sources.

## Sample Availability

Not available.
